# Correspondence

**Published:** 1870-08

**Authors:** 


### Correspondence.

### COLLEGIATE HONORS.

Canisteo, July 22, 1870.

My dear Doctor:

Having received the within business card from Philadelphia and in order to obtain "additional particulars," written immediately to H. J. Hale, M. D., 214 Jacoby Street, Philadelphia, and, as yet receiving no farther notice from him, and feeling a *peculiar disappointment* in consequence thereof, I inclose the card, thinking, perhaps, you may have been slighted by the gentleman. On the next page is a copy of my letter, and I think you will agree with me that Dr. Hale should have sent the particulars before this date, seeing I only asked him to attain just *one* degree.

This letter was designed for Dr. J. P. White, but thinking of my indebtedness to the *Journal*, I enclose $6.00, and change the direction, so you may see how a credit looks opposite my name on your book. Truly Yours,

C. P. Chamberlain.

### COLLEGIATE AGENCY.

"This Agency has been established for the purpose of giving such information as is generally necessary before entering upon a Collegiate Course of Study, or taking any of the learned Degrees."

"Books, Medicines, Instruments. &c., will also be sent C. O. D., at market rates, upon receipt of orders."

"Physician's Practices sold on accomodating terms."

"Through the recommendation of this Agency, Physicians, Lawyers, Clergymen and Teachers can obtain the Honors of all the Universities in the United States, such as the degree of A. M., A. B., M. D., S. D. D., D. D., LL.D., &c. For additional particulars, address

A. J. Hale, M. D., 214 Jacoby Street, Philadelphia.

Canisteo, Steuben Co., N. Y., July 15th, 1870.

A. J. HALE, M. D.:

*Dear Sir,*—Your card is received, and I hasten to address you for "additional particulars." I am about to change my residence to North Carolina, and should very much like to know how to attain the degree of A. M. before I make the exchange. If the Diaplome won't cost more than from $200 to $250, I think it will be a paying thing for me in my new place of residence.

Hoping to hear from you soon, I am, dear sir,

Yours Respectfully, C. P. CHAMBERLAIN.

Canisteo, July 25th, 1870.

*Dear Doctor.*—I was premature in my judgment.

The Doctor had furnished the "additional particulars."

I think, since he offers references and a guarantee of satisfaction, it will be well for me to *mcdestly* request him to furnish them before closing this rare bit of correspondence.

Inclosed herewith is his generous offer. If you do not wish any further knowledge of *my Collegiate Agent,* I will not trouble you; but I'm in for the "references," etc. What say you.

Truly, &c., C. P. CHAMBERLAIN.

Philadelphia, July 22nd, 1870.

DR. CHAMBERLAIN.

Dear Sir,—Yours of the 13th inst. received. The honorary degree of A. M. will not cost you so much as you stated.

The same can be conferred upon you, through my recommendation, for $220, all complete, and in perfect order, and sent to your address by express, C. O. D. References furnished, and satisfaction guaranteed in all cases.

When you order, your full name will be required, and such date as you desire mentioned.

Very Respectfully, A. J. HALE, M. D.

It is sufficient comment upon the rascality of the "Collegiate Agent" to publish his card. We must not omit saying that our Correspondent was unable to obtain any *"further particulars."*—ED.

